# *Robinsoniella peoriensis* Infections in Humans—A Narrative Review

**DOI:** 10.3390/antibiotics13060570

**Published:** 2024-06-20

**Authors:** Petros Ioannou, Stella Baliou, Diamantis Kofteridis

**Affiliations:** School of Medicine, University of Crete, 71003 Heraklion, Greece

**Keywords:** *Robinsoniella*, infection, prosthetic joint infection, osteomyelitis, bacteremia

## Abstract

*Robinsoniella peoriensis* is a Gram-positive, strictly anaerobic, spore-forming, rod-shaped bacterium belonging to the phylum Firmicutes and the family Lachnospiraceae. Until now, *R. peoriensis* is the only species of its genus. It was first isolated in 2003 during a study into the flora of lagoons and manure pits. Given the rarity of this microorganism and the sparse information in the literature about its way of transmission, the way to diagnose its infections and identify it in the microbiology laboratory, and its public health relevance, the present study aimed to identify all the published cases of *Robinsoniella*, describe the epidemiological, clinical, and microbiological characteristics, and provide information about its antimicrobial resistance, treatment, and outcomes. A narrative review was performed based on a Pubmed/Medline and Scopus databases search. In total, 14 studies provided data on 17 patients with infections by *Robinsoniella*. The median age of patients was 63 years and 47% were male. The most common types of infection were bone and joint infections, bacteremia, infective endocarditis, and peritonitis. The only isolated species was *R. peoriensis*, and antimicrobial resistance to clindamycin was 50%, but was 0% to the combination of piperacillin with tazobactam, aminopenicillin with a beta-lactamase inhibitor, and metronidazole which were the most commonly used antimicrobials for the treatment of these infections. The overall mortality depends on the type of infection and is notable only for bacteremia, while all other infections had an optimal outcome. Future studies should better assess these infections’ clinical and epidemiological characteristics and the mechanisms of the antimicrobial resistance of this microorganism from a mechanistic and genetic perspective.

## 1. Introduction

The identification of novel microorganisms has become more frequent in the current era, where genetic methods such as 16S rRNA gene sequencing have been implemented [1,2]. These methods have the potential to more accurately diagnose infections by microorganisms that are harder to identify by classic microbiological means [3].

*Robinsoniella peoriensis* is a Gram-positive, strictly anaerobic, spore-forming rod-shaped bacterium belonging to the phylum Firmicutes and the family Lachnospiraceae. It was first isolated in 2003 during a study into the flora of lagoons and manure pits [4]. Cotta et al. further characterized it in 2009 in terms of genetic and biochemical characteristics in the genus and species level [5]. Until now, *R. peoriensis* is the only species of the genus *Robinsoniella*.

Ever since non-clinical studies about this microorganism have been published, providing important information regarding its genomic characteristics and pathogenicity has been an aim for researchers. For example, in a recent study, McLaughlin characterized the pathogenicity of *R. peoriensis* using a pathogenomics approach. More specifically, six strains were used and allowed the conduction of the phylogenetic analysis and the identification of specific prophages inside their genome, while issues regarding its antimicrobial resistance and putative virulence factors were discussed [6]. In other studies, *R. peoriensis* has been identified in humans and animals, and, in some cases, its genome was characterized [7,8,9,10]. Several clinical reports of infections have been made in humans after the characterization of this microorganism [11,12]; however, the characteristics of infection by this rare pathogen remain largely unknown. For example, whether it is a commensal or an opportunistic pathogen, its way of transmission, the best way to diagnose its infections and identify it in the microbiology laboratory, and its public health relevance are unknown due to its rarity. Surveillance for such rare pathogens is needed since infectious diseases may emerge sporadically and seldom cause epidemics [13,14]. On the other hand, rare infectious diseases may not have been adequately recognized in cases where appropriate identification requires meticulous work or demanding procedures, such as genetic confirmation with 16s rRNA [15].

The present study aimed to identify all the published cases of *Robinsoniella* in the literature, describe the epidemiological, clinical, and microbiological characteristics, and provide information about its antimicrobial resistance, treatment, and outcomes.

## 2. Results

### 2.1. Included Studies’ Characteristics

**Figure 1:**
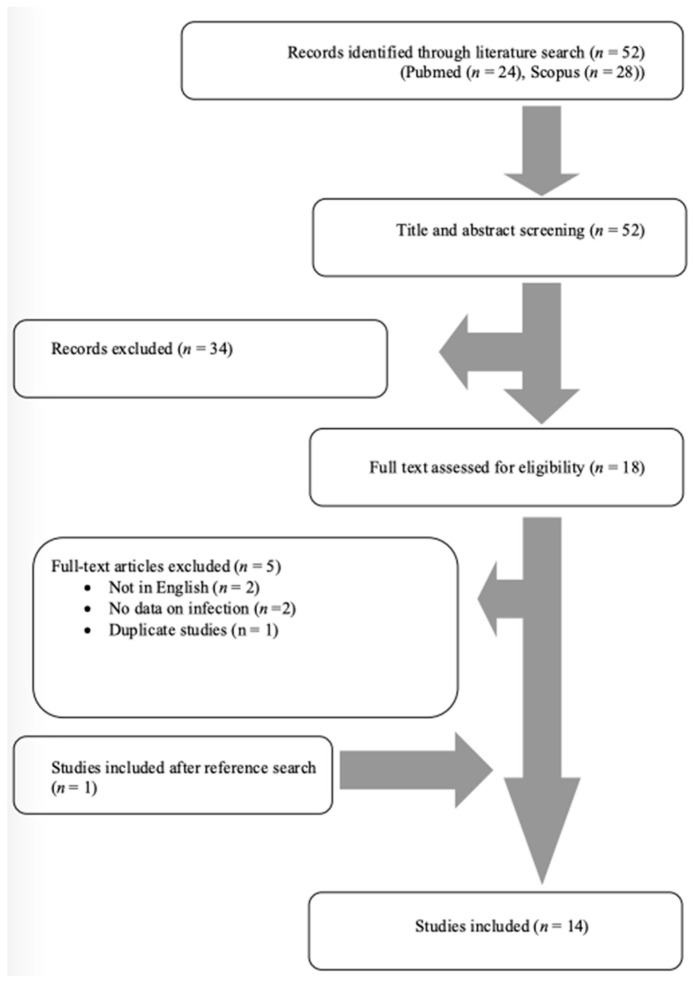
Flow diagram of study inclusion.

**Figure 2:**
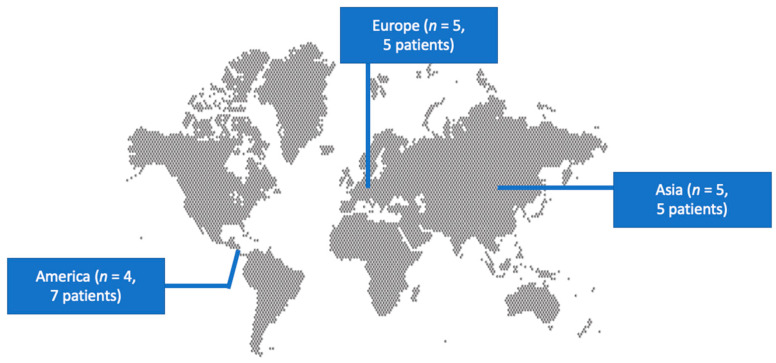
Geographical distribution of *Robinsoniella* infections worldwide.

The literature search of the PubMed and Scopus databases yielded 52 non-duplicate studies. After screening all potentially eligible studies and the snowball procedure, only 14 studies met the inclusion criteria and were considered for data extraction [11,12,16,17,18,19,20,21,22,23,24,25,26,27]. Figure 1 shows the flow diagram of study inclusion. These 14 studies provided information on 17 patients. Among them, five studies were conducted in Europe, five in Asia, and four in North and South America. There were 13 case reports and 1 case series. Figure 2 shows a graphical representation of the geographical distribution of the published cases. Table 1 shows the characteristics of the included studies in the present review.

### 2.2. Epidemiology of Robinsoniella Infections in General

The age of the patients with infection by *Robinsoniella* ranged from 3 to 84 years. The median age was 63 years, while 8 out of 17 patients (47.1%) were male. Regarding the patients’ medical history, 35.3% (six patients) had a history of surgery in the three months before the infection, with 5.9% (one patient) recovering from cardiac surgery. Notably, 35.3% (six patients) had a history of trauma and fractures requiring medical treatment and surgery. Moreover, 23.5% (four patients) had an active malignancy on chemotherapy at the time of the diagnosis of the infection by *Robinsoniella*, and 5.9% (one patient) had a cardiac implantable electronic device (CIED).

### 2.3. Microbiology and Antimicrobial Resistance of Robinsoniella Infections in General

*Robinsoniella* was isolated from the blood in 35.3% (six patients), pus in 17.6% (three patients), bone in 11.8% (two patients), surgically retrieved tissue samples in 11.8% (two patients), endometrial fluid in 5.9% (one patient), arterial homograft in 5.9% (one patient), orthopedic implant in 5.9% (one patient), and peritoneal fluid in 5.9% (one patient). The only species identified was *Robinsoniella peoriensis*. The infection was polymicrobial in 17.6% (three patients), and the other identified pathogens were *Acinetobacter* and *Enterococcus* in one patient with an osteoarticular infection, vancomycin-resistant *Enterococcus* in another patient with osteoarticular infection, and *Finegoldia magna*, *Pseudomonas aeruginosa*, *Enterococcus faecalis*, and *Enterococcus avium* in a third patient who also had pyometra with secondary bacteremia. The identification was performed based on genetic testing and, more specifically, based on 16s rRNA sequencing, in 88.2% (15 patients), and matrix-assisted laser desorption/ionization time-of-flight mass spectrometry (MALDI-TOF MS) in 23.5% (four patients—all in cases published from 2021 and on). Importantly, MALDI-TOF MS failed to identify the pathogen in 29.4% (five patients), thus requiring genetic methods to establish microbiological diagnosis. The infection was community-acquired only in 21.4% (3 out of 14 patients with the available data).

Regarding antimicrobial resistance, the E-test was the most common method used in 66.7% (8 out of 12 patients with the available data). Penicillin resistance was noted in 100% (10 out of 10 patients), resistance to quinolones was 100% (three out of three patients), resistance to clindamycin was 50% (6 out of 12 patients), resistance to metronidazole was 0% (0 out of 10 patients), resistance to aminopenicillin and the beta-lactamase inhibitor was 0% (zero out of seven patients), resistance to piperacillin and tazobactam was 0% (zero out of nine patients), resistance to vancomycin 0% (three out of three patients), and resistance to carbapenems was 0% (0 out of 12 patients).

### 2.4. Clinical Presentation of Robinsoniella Infections in General

**Figure 3:**
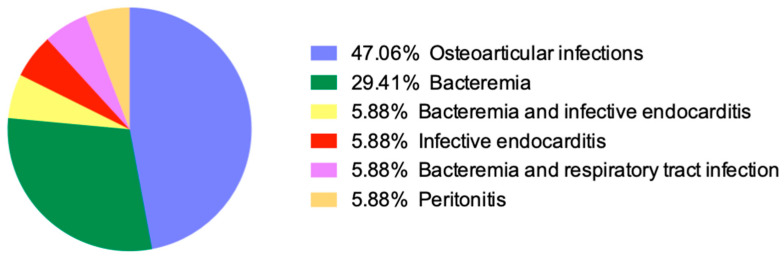
Pie chart of different types of infections caused by *Robinsoniella*.

The most common *Robinsoniella* infections were bone and joint infections in 47.1% (8 out of 17 patients), followed by bloodstream infections in 41.2% (seven patients), infective endocarditis in 11.8% (two patients), and peritonitis in 5.9% (one patient). Figure 3 shows the different type of infections by *Robinsoniella* in a pie chart. The most common clinical symptoms were fever in 66.7% (10 out of 15 patients) and sepsis in 20% (2 out of 10 patients), while a mycotic aortic aneurysm was noted in a patient with aortic homograft infection and infective endocarditis.

### 2.5. Treatment and Outcomes of Robinsoniella Infections in General

The most commonly used antimicrobials for the treatment of *Robinsoniella* infections were piperacillin and tazobactam in 40% (6 out of 15 patients), metronidazole in 26.7% (four patients), clindamycin in 20% (three patients), aminopenicillin with a beta-lactamase inhibitor in 20% (three patients), tetracycline in 13.3% (two patients), vancomycin in 13.3% (two patients), a carbapenem in 13.3% (two patients), rifampicin in 6.7% (one patient), quinolone in 6.7% (one patient), linezolid in 6.7% (one patient), a cephalosporin in 6.7% (one patient), and an aminopenicillin in 6.7% (one patient). Surgical management was performed along with antimicrobial treatment in 68.8% (11 out of 16 patients). The median treatment duration among survivors was 45 days, the range was 12 to 180 days, and the interquartile range was 16 to 68.5 days. The overall mortality was 23.5% (4 out of 17 patients) and the mortality attributed directly to the infection was 17.6% (three patients). Table 2 shows the characteristics of patients with infections by *Robinsoniella* in total and in regards to the type of infection.

### 2.6. Bone and Joint Infection Due to Robinsoniella

Eight patients with bone and joint infection by *Robinsoniella* were identified. The median age of those patients was 52 years, with a range of 3 to 74 years, an interquartile range of 21.8 to 67.8 years, and 50% (our out of eight patients) were male. Among all patients, 75% (six patients) had trauma with fractures, necessitating surgery. Half the patients had surgery within the last three months. The infection was polymicrobial in 25% (two patients) and was community-acquired in 16.7% (one out of six patients with the available data). Fever was present in 50% (three out of six patients), while no patient was septic. The median duration of treatment was 60 days, the range was 42 to 180 days, and the interquartile range was 43.5 to 128.5 days. Surgical management, along with antimicrobial treatment, was performed in all patients. No patient with a bone or joint infection due to *Robinsoniella* died.

### 2.7. Bacteremia Due to Robinsoniella

Seven patients with bacteremia by *Robinsoniella* were identified. The median age of those patients was 76 years, with a range of 42 to 84 years, an interquartile range of 47 to 79 years, and 42.9% (three out of seven patients) were male. Among all patients, 57.1% (four patients) had malignancy and were on chemotherapy, 14.3% (one patient) had undergone cardiac surgery within the last three months, and 14.3% (one patient) had a CIED. In 14.3% (one patient), a diagnosis of infective endocarditis was also made. The infection was polymicrobial in 14.3% (one patient) who also had pyometra as a source of the bacteremia. The infection was community-acquired in 16.7% (one out of six patients with the available data). Fever was present in 71.4% (five out of seven patients), while 50% (two out of four patients with the available data) were septic. The median duration of treatment among survivors was 18 days, and the range was 14 to 49 days. Surgical management as the source control, along with the antimicrobial treatment, was performed in 16.7% (one out of six patients) and, more specifically, in the patient with the pyometra. Mortality was 57.1% (four out of seven patients) and in 42.9% (three patients), mortality was directly attributed to the infection.

### 2.8. Infective Endocarditis Due to Robinsoniella

In the present analysis, two patients had infective endocarditis by *Robinsoniella*, and their ages were 70 and 79 years. One patient was male and the other was female. One of those two patients had a CIED. In only one of those two patients, bacteremia was identified. In the other patient, the diagnosis was performed with cultures of the pathogen from an excised aortic homograft and tissue cultures. Transesophageal echocardiography was needed to reach a diagnosis in both patients. The infection involved an aortic homograft in one patient, while, in the other patient, the infection involved the aortic valve and the CIED. The infection was considered community-acquired in one of those two patients. The clinical presentation was fever in the first patient and the workup led to the diagnosis of infective endocarditis along with a mycotic aneurysm, while in the second patient, it was afebrile but had an inflammatory syndrome that led to the diagnosis of the aortic valve and CIED-infective endocarditis. Surgical treatment was performed in the first patient, with the removal of the infected homograft and a mycotic aneurysm. Both patients survived and the treatment duration was 49 days for one patient, while in the other one, the duration of the treatment was not reported.

### 2.9. Peritonitis Due to Robinsoniella

Herein, one patient had peritonitis by *Robinsoniella* and was a female who was 61 years old who had recent surgery for diverticulitis and had signs of infection afterward, leading to the diagnosis of *Robinsoniella* peritonitis based on the culture of peritoneal fluid. The clinical presentation included fever. The patient was treated with antimicrobials, while the source control was performed with the percutaneous drainage of the infected fluid collection. The patient survived.

## 3. Discussion

This review summarizes the characteristics of the patients suffering from infections from *Robinsoniella* based on published studies in the literature, providing adequate information on epidemiology and mortality and data on the microbiology, clinical characteristics, and treatment. The most common types of infection were bone and joint infections, bacteremia, infective endocarditis, and peritonitis. The only identified species was *R. peoriensis*; the combination of piperacillin and tazobactam was the most commonly used antimicrobial to treat these infections. The overall mortality was high for patients with bacteremia, but all patients with other infections survived.

The identification and characterization of *Robinsoniella* infections were very recently performed [4,5]. Thus, even though some infections by this pathogen have been described in the literature, typically in the form of case reports, the exact epidemiological, microbiological, and clinical characteristics, as well as information regarding the treatment and outcomes of these infections, are yet to be clearly understood. In this review, most patients were female and the median age was 63 years. Most studies were conducted in Europe and North America. This may be highly relatable to the need for genetic studies for pathogen identification, as seen in most studies in the present review. Thus, the geographical distribution shown in the map in the present review may not represent this pathogen’s true distribution of infections. Since the pathogen was isolated from a swine-manure storage pit, it would be plausible to anticipate relevant exposure in the patients’ epidemiological history [5]. However, such exposure was not present in the reviews included in the present review. Importantly, in about half the cases, significant trauma with multiple fractures was noted in the patients’ history. This could imply that these patients were exposed to an environmental source of infection, even though the type of infection in most patients described in the present analysis was not community-acquired. Eventually, due to the very small number of patients herein and the possibility of underdiagnosis, as well as the possibility of under-reporting, safe conclusions cannot be drawn, and more studies are needed to elucidate the epidemiology of these infections.

Based on the reports included in the present review, there are three major predisposing factors in the patients’ medical histories. The first one is trauma with fractures, which necessitates orthopedic surgery. Indeed, six such patients were identified in the present review [12,21,22,24,25]. In most of these cases, trauma led to open fractures that could allow the inoculation of environmental pathogens in the patients’ bones or joints. In all these patients, surgery was performed, and this adds the possibility of exposure to hospital pathogens that could lead to hospital-acquired infections. *Robinsoniella peoriensis* is a strictly anaerobic bacterium. Infections are common after trauma, including lacerations, blunt trauma, penetrating trauma, bites, and open fractures. Most infections after trauma are mixed, being caused by aerobic and anaerobic bacteria in about 52% of cases where cultures were positive [28]. Infections only by anaerobic bacteria were found in 32% of cultures with positive results. The most common anaerobic bacteria identified in a study on such infections were *Bacteroides fragilis*, *Peptostreptococcus* spp., *Clostridium* spp., *Prevotella* spp., and *Fusobacterium* spp. [28]. Moreover, the culturing of anaerobic bacteria may have less sensitivity compared to aerobic bacteria, necessitating specific steps in the routine of a microbiology laboratory [29]. This could be associated with a reduced representation of anaerobic bacteria in the microbiology of trauma-associated infections.

Other types of surgery were also noted in the patients’ medical histories. For example, a post-operative infection occurred in a patient who had recently had diverticulitis, necessitating surgery afterward and leading to peritonitis [12]. In patients with diverticulitis and other associated intra-abdominal infections, polymicrobial infections usually occur, and they consist of aerobic and anaerobic bacteria as well [30]. The most commonly identified anaerobes in a 12-year study regarding intra-abdominal and postsurgical abdominal wound infections were *Bacteroides*, *Peptostreptococcus*, and *Clostridium* species [30]. Another patient in the present review had cardiothoracic surgery within the three months preceding the infection. In this patient, the infection caused by *Robinsoniella* was a bacteremia [12].

Finally, cancer on chemotherapy was also present in the medical history of patients with bacteremia by *Robinsoniella* [11,19,26,27]. These patients have many significant particularities. Due to the chemotherapy they are receiving, they are immunosuppressed, and this is associated with a higher likelihood of bacterial infection [31,32]. Bacterial infections in cancer patients are more commonly caused by Gram-negative bacilli, and the most commonly identified species are *Klebsiella* spp., *Pseudomonas* spp., and *Escherichia coli*. Gram-positive pathogens are also commonly involved in infections in cancer patients, with *Staphylococcus aureus* being the most common species among them [32]. Anaerobic bacteria infrequently cause bacteremia in cancer patients not receiving surgical treatment. For example, in a study by Zahar et al., 45 patients had an episode of bacteremia by anaerobic bacteria during six years at a tertiary oncologic center. Gastrointestinal and hematological malignancies were the most common diseases in the patients’ history, and the most commonly identified bacteria were *Bacteroides* spp. and *Clostridium* spp. [33]. Mortality was 14% for these patients when adequate antimicrobial treatment was provided and 63% for those who were inadequately treated. In the present review, mortality in patients with bacteremia, with about half of them being on chemotherapy due to cancer, was 43%. Thus, due to the high mortality noted in patients with bacteremia by anaerobic pathogens, anaerobic pathogens should be considered when treating patients who are at particular risk for mortality when suffering such infections, such as cancer patients, especially when oral mucositis is present or if the patient had undergone invasive procedures on the gastrointestinal tract. The empirical antimicrobial treatment should cover anaerobic bacteria, while anaerobic cultures should also be drawn along with aerobic ones [33]. Patients with cancer also have more extensive contact with the healthcare system, and this leads to an increased incidence of infections in general, as well as infections by antibiotic-resistant infections [34]. Moreover, these patients require more frequent interventions, such as the placement of central venous catheters and surgeries, and this is also associated with a higher risk of infectious complications [35].

**Figure 4:**
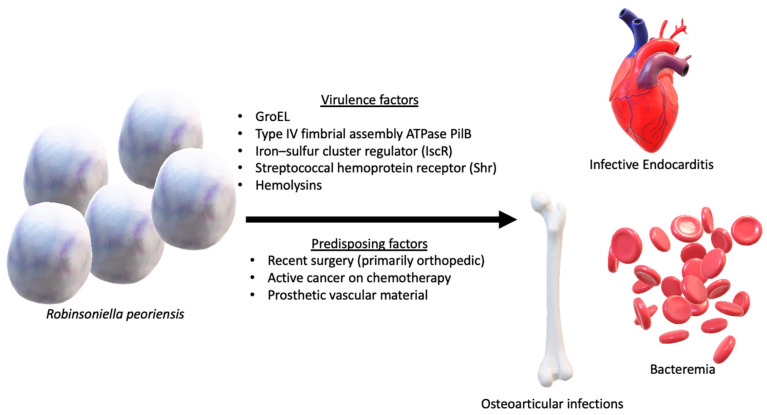
Proposed pathogenesis of *Robinsoniella peoriensis*.

The two patients with infective endocarditis by *Robinsoniella* included in the present review had bacteremia, and the other did not [20,23]. However, both patients had classic predisposing factors associated with infective endocarditis since one patient had a homograft in the ascending aorta while the other had a CIED. The workup in both patients proved that these prosthetic materials were infected, and both patients had an optimal outcome after surgery and prolonged antimicrobial treatment. Indeed, prosthetic valves, CIED, and other prosthetic materials, such as left-ventricle-assisted devices and homografts, are very well-described predisposing factors for the development of infective endocarditis in a large series of patients worldwide [36,37,38,39]. To that end, these predisposing factors are included as a minor criterion in the diagnostic criteria for the diagnosis of infective endocarditis even after the last updates of the criteria [40]. Figure 4 summarizes the possible pathogenicity of *R. peoriensis* based on the currently described virulence factors and types of infections [6].

The polymicrobial infection was relatively common in patients with an infection by *R. peoriensis*. More specifically, two patients with osteoarticular infections and one patient with pyometra and bacteremia had polymicrobial infections. This is particularly important since clinicians caring for patients with this microorganism should know the possibility of polymicrobial infections. However, whether a polymicrobial infection with *R. peoriensis* and other pathogens is associated with a higher mortality risk is unknown. In other studies, like in a Korean study or another study in critically ill patients, polymicrobial bacteremia was not associated with an increased mortality risk [41,42]. However, this is in contrast to other studies that show an increased risk for patients with polymicrobial bacteremia compared to those who had monomicrobial bacteremia [43]. Eventually, since the clinical and epidemiological characteristics of *R. peoriensis* infection are not quite clear, prospective multicenter studies for a long time will be necessary to lead to the identification of many patients with such infections and provide more data about their characteristics.

Identifying *Robinsoniella* remains difficult as most microbiology laboratories cannot access advanced molecular methods such as genetic ones. The misidentification of *Robinsoniella* based on a Gram stain and biochemical profile was very frequent. Interestingly, misidentification also occurred even when MALDI-TOF MS was used in some cases [16,18,20,21,22]. Genetic techniques such as 16s rRNA sequencing led to appropriate identification in all cases. In that direction, the use of specific 16s rRNA primers could help accurately identify *R. peoriensis* in both medical and research laboratories [15]. However, in studies published after 2021, MALDI-TOF MS was reported to have correctly identified *Robinsoniella* as the cause of infection. This is important since genetic testing is very hard to implement in everyday practice in microbiology laboratories, but MALDI-TOF MS could be more easily implemented [44]. In addition, as was recently shown in a study regarding the efficacy of MALDI-TOF MS applications in the identification of anaerobic bacteria, its accuracy may depend on the pathogen, and its accuracy for the species level was 84% [45].

There are no official antibiotic susceptibility breakpoints for *Robinsoniella*. The data in the present review are derived from case reports mentioning the pathogen’s antimicrobial susceptibility. This pathogen is often underdiagnosed, so its antimicrobial resistance patterns are of great interest. All strains with the available data were sensitive to aminopenicillin with a beta-lactamase inhibitor, piperacillin with tazobactam, carbapenems, and metronidazole. However, significant resistance to clindamycin and universal resistance to penicillin was noted. The mechanisms of antibiotic resistance have yet to be described. Antimicrobial susceptibility in anaerobic pathogens is an interesting, but difficult subject. The antimicrobial resistance of anaerobic bacteria is increasing [46,47,48,49,50]. Since the inappropriate treatment of infections by anaerobic pathogens may be associated with higher mortality, appropriate susceptibility testing practices should be performed to allow adequate treatment, especially in cases where long-term therapy is needed, in infections in specific organs, such as the central nervous system or the heart, when the infection persists despite empirical treatment, as well as in other cases [51]. Notably, even though data regarding the antimicrobial susceptibility of *R. peoriensis* have been provided in the case reports included in the present analysis, the mechanisms underlying this resistance remain unknown. Interestingly, no genes associated with antimicrobial resistance were identified by McLaughlin in his recent study on the pathogenicity of *R. peoriensis* [6]. Given that in the present review, there was notable antimicrobial resistance to some antimicrobials, the fact that no known gene associated with antimicrobial resistance was identified may imply that either the strains studied by McLaughlin would have been sensitive if tested with antimicrobial susceptibility assays, while the resistant clinical strains may have some of the known antimicrobial resistant genes, or that the resistant clinical isolates may harbor genes conferring resistance by unknown mechanisms. This underlines the need for future studies providing both in depth clinical and microbiological information to better characterize this microorganism’s antimicrobial resistance patterns that could lead to the identification of novel genes and enzymes conferring resistance to already known and used antimicrobials. This is of particular importance given the fact that genes associated with antimicrobial resistance could be transferred between pathogens if mobile genetic elements are those coding for the mediators of the antimicrobial resistance mechanism [52]. If novel genes and mechanisms of resistance are identified, important consequences in infection control may have to be implemented in the case of infection by this pathogen.

Given the above, it is reasonable that the most frequently used antimicrobials were the combination of piperacillin with tazobactam, aminopenicillin with a beta-lactamase inhibitor, and metronidazole.

This study had some notable limitations, mainly related to its narrative nature. First, the literature search may have been incomplete, and some studies may have been missed due to the search strategy. Second, the present study included only case reports and case series. The credibility of such reports depends on accurately representing the patients’ data. Finally, some of the data in the included studies were missing; thus, this study only presents and analyzes the available data.

## 4. Materials and Methods

This narrative review was performed to export data on human infection by *Robinsoniella* species. For this review, two investigators (P.I. and S.B.) independently searched PubMed/Medline and Scopus databases for eligible articles reporting “*Robinsoniella* AND infection” until 13 March 2024. Any differences between the decisions about study inclusion were resolved by consensus among the two investigators. This review only included original reports of infections from case reports and case series that provided information regarding the epidemiology, microbiology, treatment, and outcomes of *Robinsoniella* infections in humans. Only articles written in the English language were included. Reviews, systematic reviews, letters to the editor, and any other study not providing original information about these infections were excluded. Articles with no access to original data, studies presenting aggregated data, and studies referring to animals were also excluded. Moreover, studies without information on patients’ mortality and epidemiology were excluded. The references of all the included articles were also searched to assess potential studies following the snowball procedure.

Two investigators (P.I. and S.B.) used a pre-defined template to extract information from all the eligible studies in this narrative review. Data regarding age, epidemiology characteristics, the site of infection, the microbiology of species, antimicrobial susceptibility, antimicrobial treatment, and the outcomes of *Robinsoniella* infections in humans were extracted and further analyzed for this review.

## 5. Conclusions

This study provides information about the epidemiology, clinical characteristics, microbiology, antimicrobial susceptibility, treatment, and outcomes of *Robinsoniella* infections, providing important information about the potential pathogenicity of this microorganism. *R. peoriensis* was the only identified species, most commonly causing bone and joint infections in patients undergoing orthopedic surgery, more commonly due to trauma and fractures. Bacteremia, commonly in oncologic patients on chemotherapy, was the second most common type of infection. Susceptibility to the combination of piperacillin with tazobactam, aminopenicillin with a beta-lactamase inhibitor, and metronidazole was high, and these were the most commonly used antimicrobials for treatment; however, no breakpoints have been defined until now for antimicrobial susceptibility. The infection outcome mainly depended on the type of infection, which was optimal in all types except for bacteremia, which had high mortality. Future studies regarding the clinical and epidemiological characteristics of this rare pathogen, as well as studies aiming to decipher the mechanisms of its antimicrobial resistance from a mechanistic and genetic perspective, would be of great value.

## Figures and Tables

**Table 1:** Characteristics of included studies.

Author, Year	Number of Patients	Gender	Age (Years)	Type of Infection	Treatment (%)	Mortality (%)
Shen et al., 2010 [11]	1	Male	42	Bacteremia 1 (100)	Metronidazole 1 (100)	1 (100)
Gomez et al., 2011 [12]	4	1 male 3 females	45, 61, 68, 79	Bacteremia 1 (25) Peritonitis 1 (25) Bone and joint 2 (50)	Piperacillin and tazobactam 2 (50) Vancomycin 1 (25) Linezolid 1 (25) Metronidazole 1 (25) Quinolone 1 (25) Clindamycin 1 (25) NR 1 (25) Surgical management 3 (75)	1 (25)
Cassir et al., 2012 [16]	1	Female	46	Bone and joint 1 (100)	Clindamycin 1 (100) Surgical management 1 (100)	0 (0)
Jeon et al., 2012 [17]	1	Male	76	Bacteremia 1 (100)	NR	1 (100)
Rieber et al., 2016 [18]	1	Female	74	Bone and joint 1 (100)	Aminopenicillin and inhibitor 1 (100) Surgical management 1 (100)	0 (0)
Lim et al., 2017 [19]	1	Male	63	Bacteremia and lower respiratory tract 1 (100)	Carbapenem 1 (100)	1 (100)
Mertes et al., 2017 [20]	1	Male	70	Endocarditis 1 (100)	Cephalosporin 1 (100) Clindamycin 1 (100) Surgical management 1 (100)	0 (0)
Schmetterer et al., 2017 [21]	1	Female	67	Bone and joint 1 (100)	Carbapenem 1 (100) Surgical management 1 (100)	0 (0)
Schröttner et al., 2019 [22]	1	Male	58	Bone and joint 1 (100)	Aminopenicillin and inhibitor 1 (100) Piperacillin and tazobactam 1 (100) Metronidazole 1 (100) Surgical management 1 (100)	0 (0)
Ursenbach et al., 2020 [23]	1	Female	79	Bacteremia and endocarditis 1 (100)	Aminopenicillin 1 (100)	0 (0)
İnal et al., 2021 [24]	1	Male	3	Bone and joint 1 (100)	Aminopenicillin and inhibitor 1 (100) Surgical management 1 (100)	0 (0)
Krueger et al., 2022 [25]	1	Male	14	Bone and joint 1 (100)	Piperacillin and tazobactam 1 (100) Metronidazole 1 (100) Surgical management 1 (100)	0 (0)
Mejia-Gomez et al., 2022 [26]	1	Female	47	Bacteremia and pyometra 1 (100)	Piperacillin and tazobactam 1 (100) Surgical management 1 (100)	0 (0)
Furuya et al., 2023 [27]	1	Female	84	Bacteremia 1 (100)	Piperacillin and tazobactam 1 (100) Vancomycin 1 (100)	0 (0)

NR: not reported.

**Table 2:** Characteristics of the different types of infections by *Robinsoniella*.

Characteristic *	All Patients (*n* = 17)	Bacteremia ** (*n* = 7)	Bone and Joint Infection (*n* = 8)	Endocarditis (*n* = 2)	Peritonitis (*n* = 1)
Age, median in years (IQR)	63 (45.5–75)	76 (47–79)	52 (21.8–68.8)	74.5 (70–79)	61
Male, *n* (%)	8 (47.1)	3 (42.9)	4 (50)	1 (50)	0 (0)
Post-surgery (within 3 months), *n* (%)	6 (35.3)	1 (14.3)	4 (50)	0 (0)	1 (100)
Active malignancy on chemotherapy, *n* (%)	4 (23.5)	4 (57.1)	0 (0)	0 (0)	0 (0)
Fractures necessitating orthopedic surgery, *n* (%)	6 (35.3)	0 (0)	6 (75)	0 (0)	0 (0)
Polymicrobial infection, *n* (%)	3 (17.6)	1 (14.3)	2 (25)	0 (0)	0 (0)
Community-acquired, *n* (%)	3/14 (21.4)	1/6 (16.7)	1/6 (16.7)	1 (100)	0 (0)
Healthcare-associated, *n* (%)	3/14 (21.4)	1/6 (16.7)	2/6 (33.3)	0 (0)	0 (0)
Hospital-acquired, *n* (%)	7/14 (50)	3/6 (50)	3/6 (50)	0 (0)	1 (100)
Clinical characteristics					
Fever, *n* (%)	10/15 (66.7)	5 (71.4)	3/6 (50)	1 (50)	1 (100)
Sepsis, *n* (%)	2/10 (20)	2/4 (50)	0/5 (0)	0 (0)	0 (0)
Outcomes					
Overall mortality, *n* (%)	4 (23.5)	4 (57.1)	0 (0)	0 (0)	0 (0)
Infection-related mortality, *n* (%)	3 (17.6)	3 (42.9)	0 (0)	0 (0)	0 (0)

IQR: interquartile range; * denominator is the total number of patients except if otherwise mentioned, ** cases of bacteremia include one case of infective endocarditis.

## Data Availability

The data presented in this study are available on request from the corresponding author.
